# Exercise and Physical Activity eHealth in COVID-19 Pandemic: A Cross-Sectional Study of Effects on Motivations, Behavior Change Mechanisms, and Behavior

**DOI:** 10.3389/fpsyg.2021.618362

**Published:** 2021-02-22

**Authors:** Gonzalo Marchant, Flavia Bonaiuto, Marino Bonaiuto, Emma Guillet Descas

**Affiliations:** ^1^Laboratory of Vulnerabilities and Innovation in Sport, UFR STAPS, Claude Bernard University Lyon 1, Lyon, France; ^2^Faculty of Economics, Universitas Mercatorum, Rome, Italy; ^3^CIRPA – Interuniversity Research Centre in Environmental Psychology, Department of Psychology of Developmental and Socialization Processes, Sapienza University of Rome, Rome, Italy

**Keywords:** self-effcacy, intentions, attitude, digitalization, habits, automaticity

## Abstract

**Objectives:**

The aims of this research were (1) to compare the levels of physical activity of eHealth users and non-users, (2) to determine the effects of these technologies on motivations, and (3) to establish the relationship that could exist between psychological constructs and physical activity behaviors.

**Methods:**

This cross-sectional study involved 569 adults who responded to an online questionnaire during confinement in France. The questions assessed demographics, usage of eHealth for exercise and physical activity, and behavioral levels. The questionnaire also measured the constructs of Social Cognitive Theory, the Theory of Planned Behavior, and automaticity facets toward eHealth for exercise and physical activity.

**Results:**

Participants who were users of eHealth for exercise and physical activity presented significantly higher levels of vigorous physical activity and total physical activity per week than non-users (*p* < 0.001). The chi-square test showed significant interactions between psychological constructs toward eHealth (i.e., self-efficacy, behavioral attitudes, intentions, and automaticity) and physical activity levels (all interactions were *p* < 0.05). Self-efficacy was significantly and negatively correlated with walking time per week. Concerning the automaticity facets, efficiency was positive and significantly correlated with vigorous physical activity levels per week (*p* < 0.05). Then, regressions analyses showed that self-efficacy and automaticity efficiency explained 5% of the variance of walking minutes per week (ß = −0.27, *p* < 0.01) and vigorous physical activity per week (ß = 0.20, *p* < 0.05), respectively.

**Conclusion:**

This study has shown that people during confinement looked for ways to stay active through eHealth. However, we must put any technological solution into perspective. The eHealth offers possibilities to stay active, however its benefits and the psychological mechanisms affected by it remains to be demonstrated: eHealth could be adapted to each person and context.

## Introduction

The COVID-19 pandemic has led half of the world’s population to stay home or be confined to reduce the spread of the virus (Sandford, 2020). Among all the measures adopted to avoid a health crisis, many different countries affected by the virus chose to apply spatial distancing as a means to slow its spread (Abel and McQueen, 2020). This measure had three undeniable effects: the exacerbation of Internet use (Effenberger et al., 2020), the digitalization of human activity linked to health (Ting et al., 2020), and a high acceptance rate for the use of technological solutions (Ammar et al., 2020c). For this reason, the use of electronic health (eHealth) for exercise and physical activity has become an alternative for staying active during this period (Chen et al., 2020; Hammami et al., 2020). eHealth is defined broadly as the use of information and communications technology, especially the Internet, to improve or enable health and healthcare (Vandelanotte et al., 2016). In the case of exercise and physical activity, we could operationalize eHealth as digital, online, or Internet tools intended to help people for practice exercise or physical activity (e.g., an application, websites, videos, communities or social media, connected watches).

The World Health Organization (WHO) (2020), the French Ministry of Sport, and the French National Observatory of Physical Activity and Sedentary (ONAPS; Duclos et al., 2020)—as well as similar bodies in other countries – have proposed on their websites some recommendations and online platforms on how to be active during this period (e.g., Bougez-chez-Vous). Additionally, regular physical activity has been indicated as an essential factor to prevent severe complications in any future pandemic viruses similar to COVID-19 (Jakobsson et al., 2020).

Physical activity is a protective factor by directly promoting health (Jakobsson et al., 2020) and by preventing from physical inactivity as a risk factor (van der Ploeg and Hillsdon, 2017). For some authors, levels of physical inactivity could be even worse during the COVID-19 pandemic (Burtscher et al., 2020), affecting negatively all physical activity intensities (Ammar et al., 2020a). Paradoxically, confinement also became an excellent opportunity to promote physical activity. In the case of France and Italy, physical exercise was one of the rare opportunities for the population to get “out of confinement” sporadically, although limited to a few hundred meters around one’s home for an hour at a time.

The COVID-19 pandemic is unprecedented; thus, there is no information about previous users’ adoption of eHealth for exercise and physical activity during a period of confinement and what the effects are on the motivations and mechanisms of behavioral change with regards to levels of physical activity. Despite this, some elements are known regarding eHealth users under an ordinary context. Some authors suggest that the people who use them are, unsurprisingly, mainly young (Wang et al., 2016; Goodyear et al., 2019) and with a high level of education (Åkerberg et al., 2017). Although the number of people using eHealth to practice physical activity and exercise is increasing, as well as the techniques of behavioral change incorporated in these technologies, studies on the social–psychological mechanisms they influence are scarce (Hoj et al., 2017).

In the present research, we address the effect of eHealth on physical activity behaviors and mechanisms for changing the behavior of its users during confinement through the constructs of Theory of Planned Behavior (TPB; Ajzen, 1991) and Social Cognitive Theory (SCT; Bandura, 1989). These constructs are the most commonly used to underpin physical activity behaviors and eHealth (Zhao et al., 2016), and both theories have been found to predict the use of eHealth physical activity (Webb et al., 2010). Furthermore, we aim to understand if these mechanisms are related to the levels of automatic properties of habits (Bargh, 1994) during confinement. The conditions of this period could also be conducive to the development of habits based on the use of these technologies (Larose, 2015), which could be related to levels of physical activity.

### Physical Activity Levels of eHealth Users and Non-users

In terms of physical activity levels, studies show mixed results when comparing the physical activity levels of eHealth users versus non-users. On the one hand, some studies have indicated that people who use eHealth for physical activity have higher levels of physical activity than those who do not (Bort-Roig et al., 2014; Romeo et al., 2019). On the other hand, other studies have indicated that there would be no significant differences in the physical activity levels between these two groups (Milne-Ives et al., 2020). This difference could be due to the methods of measuring behaviors as well as the types of physical activity that eHealth affects. These technologies affected mainly minutes walked (Rabbi et al., 2015) and vigorous physical activity (van Drongelen et al., 2014). Although the results related to physical activity levels are unclear. Some studies suggest that people using eHealth meet the recommended levels of physical activity for health (Carroll et al., 2017).

### Psycho-Social Theories Applied to eHealth for Exercise and Physical Activity

The theoretical model of the Theory of Planned Behavior assumes that positive intentions are more likely to predict behavioral adoption than unfavorable intentions (Chatzisarantis et al., 2019). They are influenced by three factors: attitudes, subjective norm, and perceived behavioral control (Ajzen, 1991). Attitudes refer to whether the person thinks that performing a physical activity is good or bad. Subjective norm refers to people’s belief about how other people who are important to them view physical activity. Perceived behavioral control is whether people feel they can perform physical activity. This construct can be characterized by control beliefs which refer to an individual’s beliefs about the presence of factors that may facilitate or hinder the performance of the behavior (Ajzen, 2001). The results of a recent study (Herrmann and Kim, 2017) showed that eHealth usage for physical activity over 5 months appears to have a connection to usefulness (attitude) and to perceived difficulties of exercising using eHealth (perceived behavioral control). The same study showed that exercise and exercise using eHealth are not influenced by peer influence (subjective norm). Intention to exercise using eHealth had low advocacy (behavioral intention), and those who used the eHealth were more likely to have high attitude (Herrmann and Kim, 2017; Gabbiadini and Greitemeyer, 2019) and behavioral belief advocacy about the physical activity in eHealth (Hoj et al., 2017). In terms of physical activity behaviors, positive attitudes are associated with high daily walk time (Füssl et al., 2019; Gabbiadini and Greitemeyer, 2019), and positive intentions are more likely to predict physical activity levels than unfavorable intentions (Chatzisarantis et al., 2019).

The literature indicates that eHealth technologies positively influence self-efficacy, social support (Wang et al., 2019), and attitudes toward physical activity (Hosseinpour and Terlutter, 2019). Perceived self-efficacy refers to a belief in one’s own capabilities to organize and execute the courses of action required to produce given outcomes (Bandura, 1997). The use of eHealth for physical activity affects self-efficacy by including two kinds of support: individual interaction (i.e., feedback, goal settings, and reward) and social interaction (i.e., social sharing and competition) (Hosseinpour and Terlutter, 2019). The feedback, goal settings, and reward provide users with information about the progress of their actual physical activity. This information allows the individual’s reflection on their performance (Prestwich et al., 2016). Subsequently, they could increase individuals’ awareness of their real ability to perform physical activity (Harries et al., 2013; Lubans et al., 2014). As a consequence, they develop self-efficacy. It is personal success that raises their belief in possessing the capability to master physical activity (Harries et al., 2013). Another study showed that goal setting and rewards could make eHealth users confident to perform physical activity, which, in turn, also increases self-efficacy (Fukuoka et al., 2012).

### Behavior Change Mechanism of eHealth and Physical Activity Levels

The eHealth convey individuals’ impression that they can perform physical activity, and as a result, they are more likely to increase their self-efficacy and engage in more physical activity behavior (van der Weegen et al., 2015; Lewis et al., 2016). In the same vein, high self-efficacy score was associated with higher physical activity levels (Wang et al., 2019). Concerning reinforcement, Maher et al. (2014) observed that most of eHealth do not incorporate characteristics of a popular social network such as communication and emphasizing interactions. However, when these features were incorporated, the social sharing with familiar users (i.e., family, friends, or colleagues) of eHealth increases the levels of physical activity (Consolvo et al., 2006). When it comes to people who are not familiar, the results reflect a phenomenon called “awkward,” with users asking themselves “why anyone would be interested in their workout” (Ahtinen et al., 2008). Regarding sharing in social networks with familiar or strangers, users sometimes also felt disappointment when they did not receive reactions from the familiar ones and that sharing results with strangers impacted negatively their motivations toward physical activity (Munson and Consolvo, 2012). Social support was associated with high levels of physical activity (Wang et al., 2019). In confinement conditions, particularly with spatial distancing (Abel and McQueen, 2020), we could expect social support in terms of physical activities to be reduced and therefore less perceived. This condition could result in minor advocacy for reinforcement about eHealth for physical activity and present fewer levels of physical activity.

The Theory of Planned Behavior (Ajzen, 1991) and Social Cognitive Theory (Bandura, 1989) have been widely applied in studies using emerging technologies such as mobile phones and exercise applications (Gao, 2017). These theories and their constructs represent motivations and the conscious or controlled plan to enact behavior (Conner and Armitage, 1998). However, research over the last decade suggests that much of our interaction with these technologies occurs through habitual processes (Larose, 2010), which are characterized by more or less unconscious thinking (Bayer and Campbell, 2012). Habits are learned sequences of acts that have become automatic responses to specific signals and are functional in obtaining particular objectives or final states (Verplanken and Aarts, 1999). This definition implies that habits are formed through an initially intentional process that allows for the repetition of behaviors (i.e., frequency) in stable contexts (Wood and Neal, 2016), which, in turn, will lead to an increase in the automaticity of this process (Lally et al., 2010). The concept of automaticity can be understood as any cognitive process with lack of intentionality, lack of control, and lack of awareness, and it is highly efficient (Bargh, 1994). In this vein, the everyday use of mobile devices has been described as performed in a minimally conscious manner or automatically (Bayer et al., 2016). In fact, the use of eHealth could be highly influenced by the automaticity of habits, as these technologies have been incorporated into daily lives and underlying cognition (Bayer et al., 2016). This characteristic can be determined and reinforced by the cues that mobile devices emit (Larose, 2015), explaining their habitual use (Bayer and Campbell, 2012).

In terms of physical activity levels, a meta-analysis of studies on the link between habits and physical activity showed positive correlations between automaticity of habits and physical activity behavior (Gardner et al., 2011). Further research has confirmed that people with strong habits are more physically active than people with weak habit scores (Boiché et al., 2016; Rebar et al., 2016). Concerning the frequency of physical activity, behavior reflects its regularity, and it is one habit dimension (Rhodes et al., 2010).

This study represents thus an attempt to:

1. Compare the levels of physical activity of users and non-users of eHealth for exercise and physical activity,
2. Determine how these technologies affect the psychological mechanisms of eHealth users, and
3. Evaluate the relationship between these mechanisms and the levels of physical activity during the confinement period.

Based on the above-reported literature, the following leading group of hypotheses can be stated:

H1: We expected no difference of physical activity levels between eHealth users and non-users during confinement.

H2: We hypothesize that eHealth users would increase perceived self-efficacy and attitudes toward physical activity.

H3: We hypothesize that self-efficacy and attitudes would be positively associated with physical activity levels.

H4: Finally, similar to previous work, we expect a positive relationship between automaticity of habit levels (Larose, 2015) toward eHealth and the levels of physical activity behavior (Gardner et al., 2011; Boiché et al., 2016).

## Materials and Methods

### Recruitment

The study sample comprised of respondents who were recruited through Drag n Survey^®^, posting invitations in social media (Facebook, Instagram, and LinkedIn), and university website. Drag n Survey is an online questionnaire provider that allows one to develop and customize questionnaires according to the type of study. The sample was limited to respondents who were 18 years or older and residents in France during confinement. The global results will be sent to the participants who completed the questionnaire as an incentive. In order to prevent that a single user fills in the same questionnaire multiple times, only one response per IP address was possible. The completion or internal consistency of specific (or all) items was enforced using server-side techniques (i.e., after submission displaying the questionnaire and highlighting mandatory but unanswered items). According to Baruch and Holtom (2008), the average level of response rate of Internet survey for social sciences, to be acceptable, is approximately 53%.

### Ethics Statement

The Institution Ethics and Review Board approved the study (2218023v0-CNIL), and it was carried out by the French methodological reference MR-001. This reference indicates that each participant must be informed of the purpose of the research. This statement was in accordance with the Declaration of Helsinki, and the participants were requested to be honest and as accurate as possible in their responses. The duration of the survey was clarified. The participants were assured that the results would be used only for this study and that their privacy would be guaranteed. If the participants did not want to participate in the survey, they could turn off the electronic questionnaire and drop out. If the questionnaire was completed and submitted, the participant was considered to have provided informed consent. Only those who voluntarily agreed to participate in the survey were included in the research.

### Procedure

An electronic survey constructed through Drag n Survey^®^ web-based software was used to collect data. The survey was available in Drag n Survey server from 24th of April 2020 to 10th of May 2020 (the end of French confinement during COVID-19 first pandemic wave). A total of 602 respondents visited the website of the study. The survey gathered information regarding self-report measures of Theory of Planned Behavior, Social Cognitive Theory, automaticity, and self-reported physical activity behavior.

### Sample

The final sample (*N* = 569) comprised of 64.1% women. The participants were between 18 and 73 years of age (*M* = 31.89, SD = 13.59).

### Measures

Demographic information was gathered, and the respondents were asked to report their age, gender, and the highest level of education obtained (Table 1). Additional information relating to eHealth usage was also gathered. The participants indicated if they were users or non-users of eHealth physical activity tools (i.e., “When we say ‘online’ or ‘the Internet,’ we are referring to content that you use regularly”), for example, WhatsApp or Snapchat contacts, an Application, websites like YouTube, online communities or social media. Then, the participants answered if they use an eHealth to practice physical activity/exercise by answering yes or no. The participants were also asked to choose what eHealth they use to practice a physical activity/exercise. The options were: Application, Website, Internet videos, and others. The next question was about when did they start using this eHealth tool for physical activity/exercise/sport (i.e., before confinement or during confinement) and how many times per week. We then asked about how many eHealth tools they used to practice physical activity before and during confinement.

**TABLE 1:** Sample characteristics.

**Demographics**	
**Age (*n* = 569), mean (SD)**	31.89 (13.59)
18–25	254(44.6%)
26–34	103(18.1%)
35–54	139(24.4%)
55–64	42(7.4%)
65 or older	31(5.4%)
**Gender (*n* = 548)**	
Male	197(36%)
Female	351(64%)
**Education (*n* = 541)**	
Primary school	51(9%)
Secondary	149(28%)
University	341(63%)
**eHealth user for physical activity (*n* = 513)**	
User	299(58%)
No-User	214(42%)
**Type of eHealth for practice physical activity and exercise (*n* = 299)**	
Application	123(41%)
Website	56(19%)
Videos	117(39%)
No answer	3(1%)
**Starting to use a eHealth for physical activity (*n* = 290)**	
Before the lockdown	129(45.5%)
During the lockdown	161(55.5%)

The participants were also asked to name their favorite eHealth tool for physical activity/exercise/sport during confinement, what was the price, and how did they find out about the eHealth tool they currently use to practice physical activity during confinement.

#### Instruments

The eHealth to practice physical activity was defined in this section as follows: “We focus on digital, online, or Internet tools.” When we say “online” or “the internet,” we are referring to eHealth that you use regularly to practice physical activity or exercise (for example, WhatsApp or Snapchat contacts, an Application, websites like YouTube, online communities or social media, connected watches).

The Social Cognitive Theory and Theory of Planned Behavior items had been translated especially for this research without any previous validation. They were adapted from English to French, following a reverse translation (Brislin, 1986). First, the scale was translated by two bilingual people from English to French. Then, two other bilingual persons translated it from French to English to analyze the degree of coincidence with the wording and the meaning of the original items. Then, this sequence was repeated by a linguist and a professional translator. After that, it was verified as to whether the original sense of the scale had been maintained. Finally, the French format of the scales was drafted.

##### Theory of planned behavior constructs

###### Attitudes

Attitudes were assessed for eHealth use toward physical activity with five items to tap the instrumental aspect of attitude as suggested by Ajzen (2001). The first was “Using the eHealth for physical activity has increased (my motivation to be physically active) during the confinement.” The second was “Using the eHealth for physical activity has increased (my attitudes about the importance of physical activity in preventing disease) during the confinement.” The third was “Using the eHealth for physical activity has increased (my desire to be physically active) during the confinement.” The fourth was “Using the eHealth for physical activity has increased (my desire to be healthy) during the confinement.” The fifth was “Using the eHealth for physical activity has increased (my motivation to set goals to be physically active) during the confinement.” All items were scored on a five-point scale from strongly disagree (1) to (5) strongly agree. The internal consistency for the five items was acceptable (α = 0.80).

###### Subjective norms

Subjective norms were measured by one item using the same five-point Likert scale from strongly disagree (1) to (5) strongly agree “Using the eHealth for physical activity has increased (my belief that people important to me want me to be physically active) during the confinement.” This component was a single item from a scale based on the findings of Rhodes and colleagues (Rhodes et al., 2006).

###### Behavioral belief

Behavioral belief was measured with four items for eHealth use toward physical activity and standard to the Theory of Planned Behavior (Ajzen, 2002). The first item was “Using the eHealth for physical activity has increased (my belief that physical activity can prevent disease) during the confinement.” The second item asked was “Using the eHealth for physical activity has increased (my belief that physical activity is important in preventing disease). The third was “Using the eHealth for physical activity has increased (my belief that physical inactivity leads to disease) during the confinement.” The fourth item was “Using the eHealth for physical activity has increased (my belief that diseases related to physical inactivity are harmful) during the confinement.” All items were scored on a five-point scale from strongly disagree (1) to (5) strongly agree. The reliability of the four items was good (α = 0.88).

###### Intention

Intention was assessed by one item recommended by Courneya and McAuley (1994): “Using the eHealth for physical activity has increased (my intentions to be physically active) during the confinement.” The item was scored on a five-point scale from strongly disagree (1) to (5) strongly agree. This item was used to create the intention–behavior profiles because it has demonstrated excellent test–retest reliability and predictive validity as a single-item measure of intention (Courneya and McAuley, 1994; Rhodes and Courneya, 2003).

##### Social cognitive theory constructs

###### Self-efficacy

Three items measured self-efficacy. The first was “Using the eHealth for physical activity has increased (my ability to be physically active).” The second was “Using the eHealth for physical activity has increased (my confidence that I can be physically active).” Finally, the third was “Using the eHealth for physical activity has increased (my ability to achieve my physical activity goals) during the confinement.” The reliability was acceptable (α = 0.77).

###### Subjective knowledge

Three items assessed subjective knowledge. The first item was “Using the eHealth for physical activity has increased (my knowledge of ways in which I can be physically active) during the confinement.” The second item was “Using the eHealth for physical activity has increased (my knowledge of the diseases that are caused by physical inactivity).” The third was “Using the eHealth for physical activity has increased (my knowledge of the benefits of being physically active) during the confinement.” All items were scored on a five-point scale from strongly disagree (1) to (5) strongly agree. The internal consistency was adequate (α = 0.69).

###### Reinforcement

Two items measured reinforcement. The first item was “Using the eHealth for physical activity has increased (the social support I have received for being physically active).” The second item was “Using the eHealth for physical activity has increased (the positive feedback I have received for being physically active) during the confinement.” The two items were scored on a five-point scale from strongly disagree (1) to (5) strongly agree. The Pearson *r* value was good (*r* = 0.75).

###### Automaticity

Automaticity was measured by nine items of the Generic Multifaceted Automaticity Scale, a validated scale in French (Boiché et al., 2016). This instrument assesses three dimensions of automaticity—lack of intentionality, lack of control, and efficiency—with three items for each one. The nine items were scored on a five-point scale from 1 (strongly disagree) to 5 (strongly agree). The internal consistency of the nine items was acceptable (α = 0.78).

###### Lack of intentionality

Three items measured lack of intentionality. The first item was “To use my app for practicing my physical activity is something (that I use instinctively, no need to mark it down in my agenda) during the confinement.” The second was “To use my app for practicing my physical activity is something (that I use without having to think about it before) during the confinement.” The third was “To use my app for practicing my physical activity is something (about which I do not wonder whether I am going to use it or not, I just use it) during the confinement.” The measure showed borderline adequate internal consistency (α = 0.64).

###### Lack of control

Three items assessed lack of control. The first item was “To use my app for practicing my physical activity is something (I would find hard not to use it) during the confinement.” The second was “To use my app for practicing my physical activity is something (that would require effort not to use it) during the confinement.” The third was “To use my app for practicing my physical activity is something (that makes me feel weird if I do not use it) during the confinement.” The reliability was borderline adequate (α = 0.66).

###### Efficiency

Efficiency was assessed with three items. The first item was “To use my app for practicing my physical activity is something (on which I do not have to focus to use it properly) during the confinement.” The second item was “To use my app for practicing my physical activity is something (that I could use “eyes closed” once I’m started) during the confinement.” The third item was “To use my app for practicing my physical activity is something (that I can use in ‘automatic pilot’) during the confinement.” The reliability was acceptable (α = 0.72).

###### Physical activity behavior

Physical activity behavior was measured using the International Physical Activity Short Form (IPAQ-SF; Craig et al., 2003). Three types of physical activity were assessed—walking, moderate activity, and vigorous activity—as sitting time as well. This instrument is considered to estimate the total physical activity in minutes per week at metabolic equivalent (MET) and time spent sitting. For example, for the vigorous physical activities, the items were “During the last 7 days, on how many days did you do vigorous physical activities like heavy lifting, digging, aerobics, or fast bicycling?” and “How much time did you usually spend doing vigorous physical activities on one of those days?” For each of these four types of activities, the subjects were also asked to estimate the total time (in hours and/or minutes) spent doing that activity in the past week.

### Data Analyses

First, the analysis of the data included the identification of the users and non-users of eHealth for physical activity. The next step was to classify the participants into active or inactive according to WHO recommendations for levels of physical activity for health in adults (i.e., 150 min of moderate to vigorous physical activity per week). Student’s *t*-tests were calculated to determine if there were differences between the physical activity levels of eHealth users and non-users. The chi-square was also calculated to determine the number of eHealth users and non-users who reached the physical activity levels recommended for health.

Then, in order to evaluate how eHealth affects the psychological constructs (i.e., TPB, SCT, automaticity facets), the averages and trends of the scores were analyzed, comparing each construct by Student’s *t*-test.

Next, to evaluate the relationship between psychological constructs and physical activity levels, two categorizations were carried out. The first was a symmetry/asymmetry analysis of the psychological constructs of the Theory of Planned Behavior, Social Cognitive Theory, and automaticity. We divided each variable of the psychological constructs of the Theory of Planned Behavior, Social Cognitive Theory, and automaticity into three groups: low advocacy (< 2, disagreement response options), ambivalence (> 1.9 and ≤ 3.9, neither disagreement nor agreement response options), and high advocacy (> 4; agreement response options). These variable categorizations were done purposefully using absolute values (i.e., not simple median splits) to examine symmetry across scale responses. The second was classifying the subjects into three groups on the basis of three IPAQ profile groups. The first group corresponds to the high level of physical activity that reaches (a) vigorous-intensity activity on at least 3 days (20 min minimum, achieving a minimum total physical activity of at least 1,500 MET minutes/week) or (b) seven or more days of any combination of walking and moderate-intensity or vigorous-intensity activities, achieving a minimum total physical activity of at least 3,000 MET minutes/week. The second group was moderate, in which individuals reached (a) three or more days of vigorous-intensity activity of at least 20 min per day or (b) five or more days of moderate-intensity activity and/or walking of at least 30 min per day or (c) 5 or more days of any combination of walking, moderate-intensity activities, or vigorous-intensity activities, achieving a minimum total physical activity of at least 600 MET minutes/week. The third group is called low, where individuals did not reach the levels described above.

After that, a series of chi-square analyses was calculated to test the independence between the advocacy of psychological constructs and IPAQ physical activity profiles (i.e., low, moderate, and high).

Finally, a correlation analysis was calculated to determine the relationship between psychological constructs and physical activity levels. Multiple regressions analysis were also calculated with physical activity in MET minutes per week as dependent variable and psychological constructs (i.e., SCT, TPB, and automaticity) and frequency of behaviors as independent variables.

Statistical significance was set at *p* < 0.05 in all the analyses.

## Results

### Preliminary Analyses

Concerning the usage of eHealth for physical activity and exercise, 58.3% (299/569) of the respondents reported being users. These participants used the eHealth at least three times per week (*M* = 3.77, SD = 2.12), were significantly younger (*M* = 29.88 years old, SD = 12.13) than non-users (*M* = 35.37 years old, SD = 15.06), *t*(511) = 4.40, *p* < 0.001. From these respondents, 55.7% started to use the eHealth during confinement. The types of eHealth for physical activity used in confinement were applications = 42% (125/299), Internet videos = 39% (118/299), and websites = 19% (56/299). Only 36% (108/299) of the respondents reported using a single physical activity eHealth, whereas 19.3% (58/299) reported using two physical activity eHealth and 8% (24/299) had three physical activity eHealth. There were 63 different applications reported by the study respondents, and most were free. The sources of information about the eHealth tool for physical activity were as follows: Internet = 125 (22%), word of mouth = 116 (20.4%), advertising = 18 (3.2%), mail (0.2%), others = 12 (2.1%), television = 5 (0.9%), and social networks = 3 (0.5%). Table 1 presents study sample descriptive statistics. There were no significant differences (*p* > 0.05) between participants in terms of confinement days (*M* = 46.58, SD = 4.2) for the variables of interest.

### Physical Activity Levels of eHealth Users Versus Non-users

**FIGURE 1:**
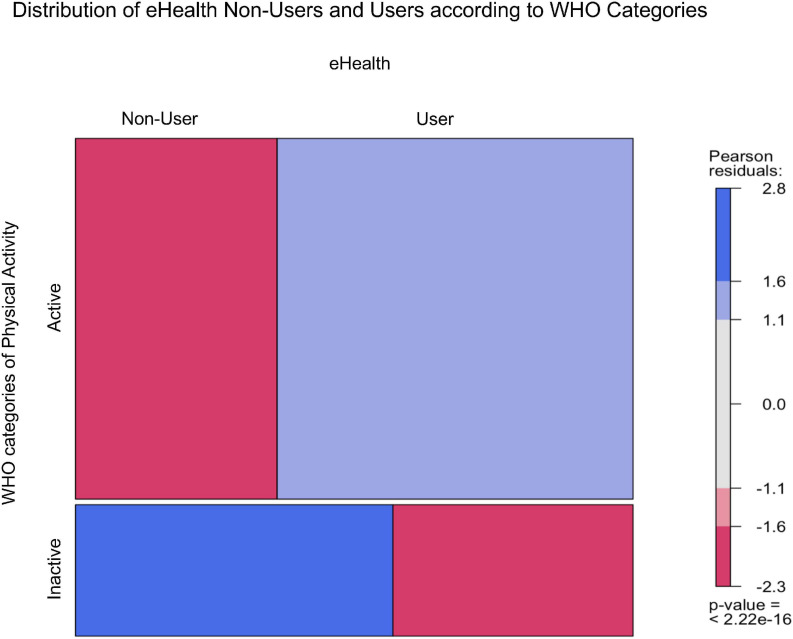
Distribution of eHealth users and non-users according to WHO categories of physical activity.

Table 2 presents the comparison of physical activity levels between eHealth users and non-users. The *t*-test analysis showed that eHealth users practiced significantly more MET minutes of vigorous physical activity per week (*M* = 3,799.17, SD = 3,415.61) than non-users (*M* = 2,343.42, SD = 2,849.54), *t*(407) = −4.6, *p* < 0.001. The eHealth users also presented significantly more MET minutes of total physical activity per week (*M* = 6,202.76, SD = 4,750.03) than non-users (*M* = 4,745.17, SD = 4383.55), *t*(407) = −3.2, *p* = 0.001. According to WHO recommendations for physical activity, eHealth users were more likely to be classified as active (80 versus 63%) than non-users [*X*^2^(2) = 17.8, *p* < 0.001]. Figure 1 shows that there are significantly more eHealth users classified as active than non-users. The widths of the boxes are proportional to the percentage of non-users (42%) and users (58%), respectively. The heights of the boxes are proportional to the percentage of people who were classified as active and inactive. Among the people classified as active, 64% were users and 36% were non-users.

**TABLE 2:** Comparison of physical activity levels of non-users and users of eHealth.

	**Non-users (*n* = 196)**		**Users (*n* = 213)**		**95% CI for mean low, high**	***p***	***t***	***df***
**Outcome IPAQ-SF**	***M***	**SD**	***M***	**SD**				
vPA MET/week	2,343.42	2,849.54	3,799.17	3,415.61	−2,070.03, −841.45	0.000	−4.65	407
mPA MET/week	1,625.51	1,704.62	1,604.56	1,698.48	−3,10.06, 352.04	0.901	0.12	407
Walk MET/week	776.173	1,267.09	799.00	1,261.66	−2,68.82, 223.16	0.855	−0.18	407
Total MET/week	4,745.17	4,383.55	6,202.76	4,750.03	−2,348.36, −566.80	0.001	−3.21	407

### The eHealth Usage Effect on Perceived Self-Efficacy and Attitudes Toward Physical Activity

In terms of SCT, perceived self-efficacy scored (*M* = 3.66, SD = 0.91) significantly (*p* < 0.001) higher than reinforcement (*M* = 2.87, SD = 1.10) and knowledge (*M* = 3.31, SD = 0.93). For the TPB constructs, intention scores (*M* = 3.94, SD = 1.02) were significantly higher than behavioral attitudes (*M* = 3.75, SD = 0.81), behavioral beliefs (*M* = 3.49, SD = 1.04) and subjective norms (*M* = 2.87, SD = 1.32), *p* < 0.001. These results showed that descriptive scores of self-efficacy, behavioral attitude, and intention had medium–strong mean score. Knowledge, reinforcement, and subjective norms had a mean of around the center of the scale, suggesting ambivalence.

### Relationship Between Psychological Constructs and Physical Activity

**FIGURE 2:**
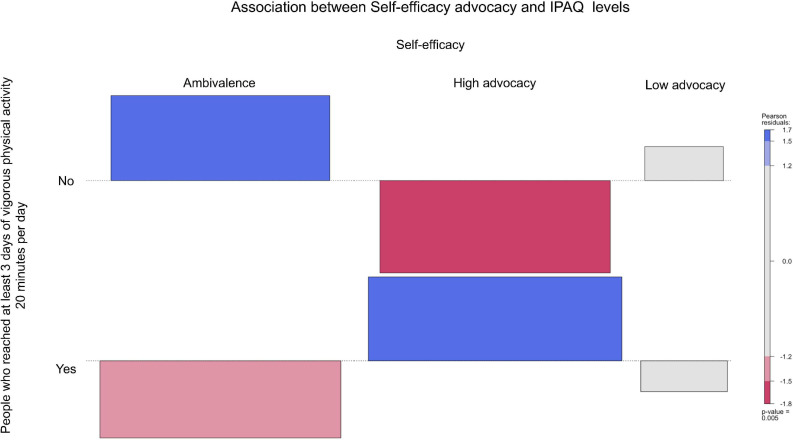
Self-efficacy advocacy of people who reached at least 3 days of vigorous physical activity 20 min per day.

**FIGURE 3:**
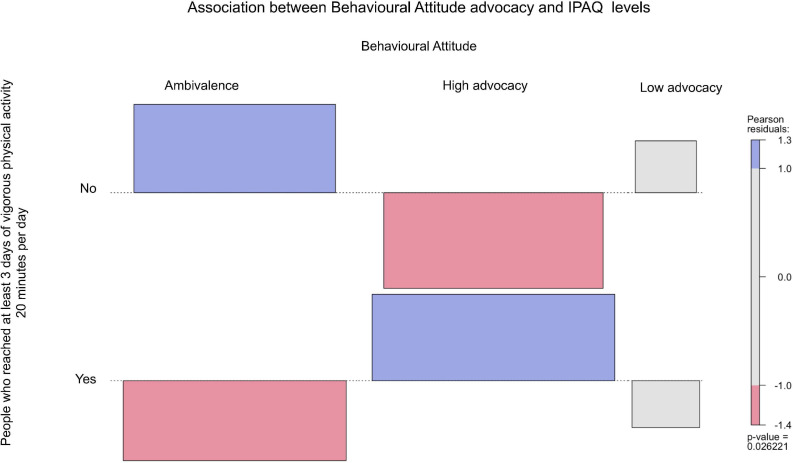
Behavioral attitude advocacy of people who reached at least 3 days of vigorous physical activity 20 min per day.

**FIGURE 4:**
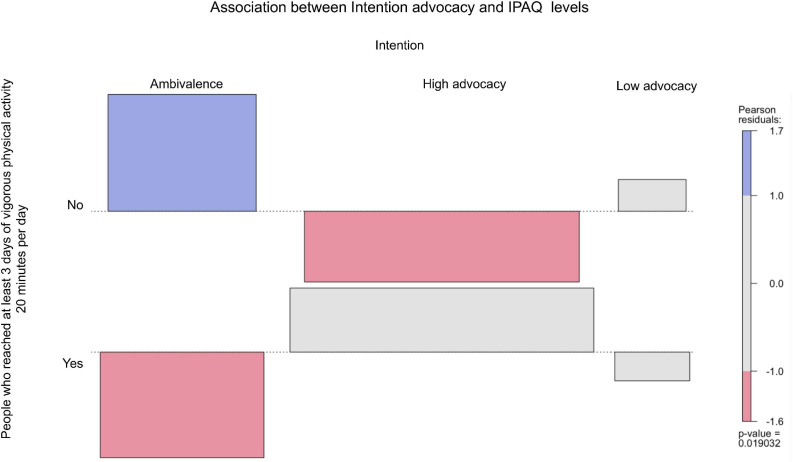
Intention advocacy of people who reached at least 3 days of vigorous physical activity 20 min per day.

**FIGURE 5:**
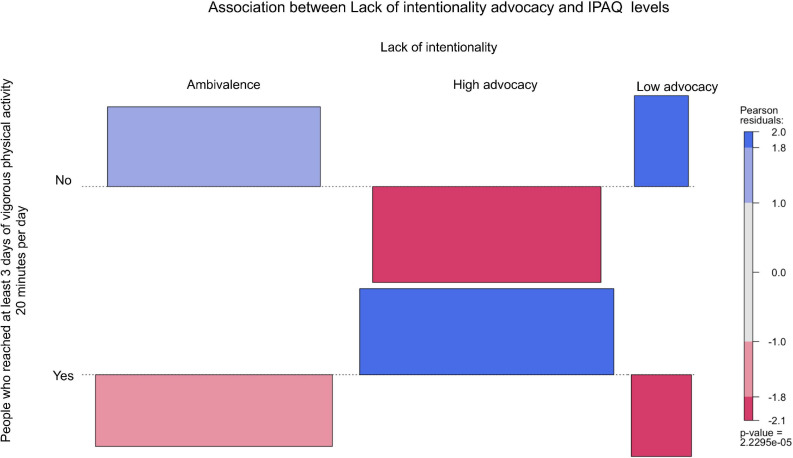
Lack of intentionality advocacy of people who reached at least 3 days of vigorous physical activity 20 min per day.

**FIGURE 6:**
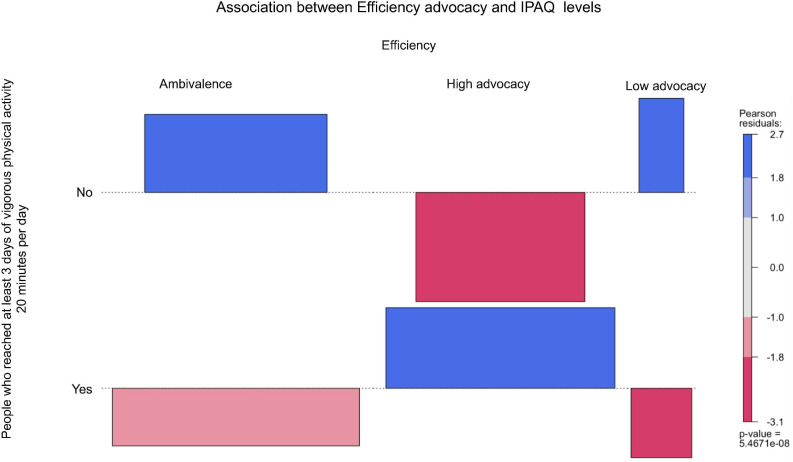
Efficiency advocacy of people who reached at least 3 days of vigorous physical activity 20 min per day.

From Figures 2–6, when the observed frequency of a cell was higher than expected, the box rises above the baseline; otherwise, the box falls below the baseline. Figure 2 shows that there were significant interactions [*X*^2^(2) = 11.17, *p* = 0.004] between high self-efficacy advocacy and one vigorous physical activity profile (i.e., ≥ 3 days of vigorous physical activity ≥ 20 min/day). High self-efficacy advocacy and people who reached at least 3 days of vigorous physical activity during 20 min per day were significantly and positively associated (*p* = 0.005). Figure 3 shows that the association of high advocacy of behavioral attitude and people who reached at least 3 days of vigorous physical activity 20 min per day was statistically significant [*X*^2^ (2) = 7.28, *p* = 0.026)]. Figure 4 shows a significant interaction between intention advocacy and ≥ 3 days of vigorous physical activity ≥ 20 min/day [*X*^2^(2) = 6.92, *p* = 0.031]. Users of eHealth for physical activity who present high advocacy for self-efficacy and attitudes were more likely to reach ≥ 3 days of vigorous physical activity during ≥ 20 min a day than those with ambivalence or low advocacy for those psychological constructs. Concerning automaticity facets and users who reached ≥ 3 days of vigorous physical activity during ≥ 20 min per day, a significant interaction was found for lack of intentionality advocacy [*X*^2^(2) = 22.34, *p* < 0.001] and efficiency advocacy [*X*^2^(2) = 34.50, *p* < 0.001]. Figures 5, 6 indicate that there are significantly more individuals with high lack of intentionality and high efficiency advocacy reaching at least 3 days of vigorous physical activity during 20 min per day.

As can be seen in Table 3, only two psychological constructs, self-efficacy and efficiency, were associated with the physical activity levels. Self-efficacy was significantly and negatively correlated with walking MET minutes per week. Concerning the automaticity facets, there was a positive and significant correlation between efficiency and vigorous physical activity MET per week. The eHealth frequency usage was positive and significantly associated with the automaticity facets, vigorous physical activity MET minutes per week, and total physical activity MET minutes per week.

**TABLE 3:** Descriptive and correlations of physical activity, frequency of eHealth, Social Cognitive Theory, Theory of Planned Behavior, and automaticity constructs.

	**5**	**6**	**7**	**8**	**9**	**10**	**11**	**12**	**13**	**14**	**15**	***M***	**SD**
vMET—min/week	0.03	0.04	0.02	0.07	0.07	–0.07	0.04	0.13	0.02	0.21**	0.36**	3,140.42	3,237.01
mMET—min/week	0.00	–0.00	0.01	–0.04	–0.08	–0.02	0.01	–0.08	–0.09	0.01	0.11	1,603.49	1,697.54
Walk MET—min/week	−0.19**	–0.02	0.05	–0.10	–0.02	–0.05	–0.11	–0.06	–0.06	–0.07	0.01	776.93	1,252.80
Total MET—min/week	–0.02	0.02	0.03	0.01	0.02	–0.07	0.00	0.04	–0.03	0.14	0.31**	5,520.87	4,601.00
Self-efficacy	1	0.57**	0.28**	0.79**	0.42**	0.22**	0.73**	0.38**	0.30**	0.16*	0.19*	3.66	0.91
Knowledge	1	0.39**	0.69**	0.66**	0.34**	0.48**	0.23**	0.22**	0.06	0.13	2.87	1.10	
Reinforcement		1	0.42**	0.33**	0.40**	0.36**	0.19*	0.16*	0.13	0.04	3.31	0.93	
Behavioral attitude		1	0.62**	0.31**	0.78**	0.25**	0.21**	0.06	0.10	3.75	0.81		
Behavioral belief			1	0.43**	0.35**	0.20**	0.22**	0.11	0.02	3.49	1.04		
Subjective norms			1	0.16*	0.09	0.24**	0.00	–0.04	2.87	1.32			
Intention				1	0.32**	0.22**	0.11	0.09	3.94	1.02			
Lack of intentionality				1	0.38**	0.49**	0.23**	3.75	0.83				
Lack of control					1	0.29**	0.27**	2.70	0.89				
Efficacy					1	0.25**	3.58	0.99					
eHealth frequency (times/week)						1	3.71	2.19					

vMET = vigorous physical activity; mMET, moderate physical activity. *p < 0.05, **p < 0.01.

### Association Between Physical Activity Levels, Psychological Constructs, and eHealth Frequency

**FIGURE 7:**
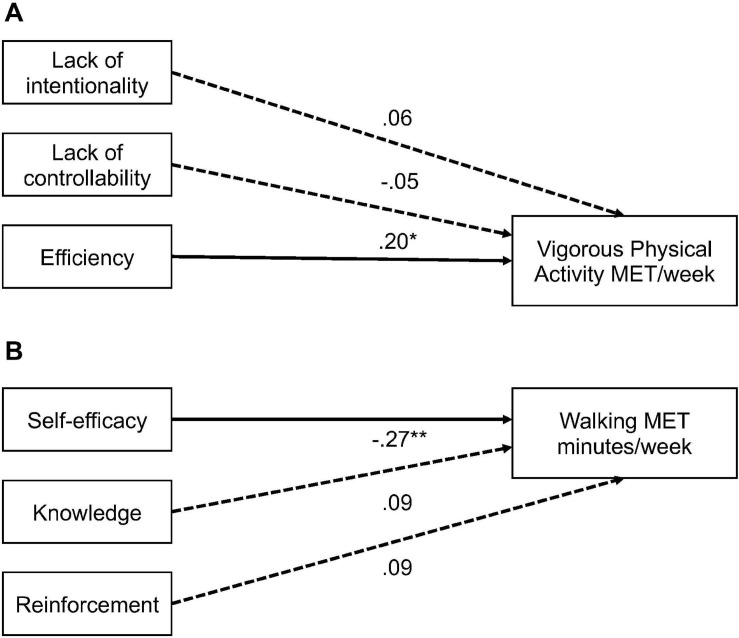
The path **(A)** shows association between automaticity facets and vigorous physical activity MET per week. The path **(B)** shows association between social cognitive theory constructs and walking minutes MET per week. *p < 0.05, **p < 0.01.

Figure 7 highlights the associations between physical activity levels, Social Cognitive Theory, and automaticity facets. Self-efficacy was negatively and significantly associated to walking MET minutes per week (ß = −0.27, *p* < 0.01), explaining 5% of the variance [*F*(3, 168) = 3.34, *p* < 0.02, *R*^2^ = 0.05, *R*^2^-adjusted = 0.04]. As far as the concepts of the Theory of Planned Behavior are concerned (i.e., attitudes, behavioral belief, subjective norms, and intention), regressions showed that none of them were associated with physical activity levels related to eHealth use. For the automaticity facets, the efficiency was positively and significantly associated with vigorous MET minutes per week (ß = 0.20, *p* = 0.021), explaining 5% of the variance [*F*(3, 175) = 3.20, *p* < 0.03, *R*^2^ = 0.05, *R*^2^-adjusted = 0.04). Simple linear regressions showed that frequency of eHealth usage predicted self-efficacy (ß = 0.15, *p* = 0.016), behavioral attitude (ß = 0.14, *p* = 0.040), and automatic properties of eHealth usage (i.e., lack of intentionality: ß = 0.22, *p* = 0.001; lack of control: ß = 0.32, *p* < 0.001; and efficiency: ß = 0.28, *p* < 0.001).

## Discussion

The context of the COVID-19 pandemic led many countries to implement containment. This context increased the use of the Internet and, in particular, the pursuit of eHealth-related physical activity (ReportLinker, 2020). For that reason, this study had three objectives: the first was to see if eHealth use impacted physical activity levels; the second was to assess the impact of eHealth use on the psychological mechanisms in a COVID-19 pandemic context; and, third, to explore what was the relationship between those mechanisms and the levels of physical activity assessed by questionnaires.

The first hypothesis was that there would be no difference in physical activity levels between eHealth users and non-users for physical activity during confinement. This hypothesis was partially supported. There were no differences in levels of walking and neither in moderate physical activity, but there were differences in vigorous physical activities and total levels. A consequence of the use of eHealth in the practice of physical activity is that it would positively impact only vigorous physical activity, confirming previous findings (van Drongelen et al., 2014). A possible explanation would be that the specific context of the confinement limited physical activity practice in terms of the time (i.e., 1 h) and the area (e.g., alone, within a maximum radius of 1 km from home), which could translate into similar levels of physical activities as far as moderate intensity (e.g., walking, running) is concerned. Another reason that may explain this difference in vigorous physical activity is the type of eHealth used (i.e., health and fitness).

In general, these e-health applications involve vigorous, short-term physical activity, and this can be done in small spaces (e.g., the living room of an apartment or a balcony). For this reason, it is likely that users were more numerous in reaching the recommended levels of physical activity for health than non-users. These results confirmed that eHealth users outnumbered non-users in reaching these levels (Carroll et al., 2017).

The second hypothesis corresponded to a group of hypotheses on the influence of the use of eHealth on the psychological mechanisms of behavior change (i.e., SCT, TPB, and automaticity). In our study, one of the mechanisms positively affected by the use of eHealth for physical activities was self-efficacy. One of our hypotheses indicated that eHealth users would present high levels of this psychological construct (Hoj et al., 2017). The eHealth technologies for physical activity use feedback, goal setting, and reward as behavior change techniques that directly affect self-efficacy (Hosseinpour and Terlutter, 2019). Thus, as people use eHealth for physical activity, this makes them increasingly aware of their results, and as a consequence, this could increase the perception of their ability to perform the exercises proposed in them (Harries et al., 2013). However, self-efficacy also could be negatively affected if people do not achieve their goals or if the goals proposed by eHealth are too high, either in terms of technical difficulty or physical activity intensities. The differences in self-efficacy are associated with variations in skill level, perceptions influenced by personality, motivation, and the task itself.

In contrast, the other variables of Social Cognitive Theory incorporated in this study, knowledge and reinforcement, presented lower levels than self-efficacy. These results could be analyzed from two perspectives. The first would be considering that the eHealth for exercise and physical activity would be focusing mainly on the instructions to execute the exercises (Conroy et al., 2014), for example, the position of the body and the type of movement to complete the exercise. When these tools present more information, there is an idea of the physical capabilities worked on (e.g., strength, flexibility, aerobic capacity). Thus, the second angle of analysis would have to consider the neglect of information that might be relevant in guiding people in understanding the benefits of staying active and in reducing the risks associated with physical inactivity. The literature shows that people who know the benefits of physical activity tend to be more active (Fredriksson et al., 2018). However, recent studies show that this knowledge represents a basic understanding that physical activity is “good” for health (Fredriksson et al., 2018), and even when knowledge about the benefits of physical activity and the participation requirements for achieving those benefits are increased, this does not necessarily represent increased motivation to engage in physical activity (Segar et al., 2020).

For this reason, one option for improving this information could take into account the four levels of knowledge proposed by Chapman and Liberman (2005) and adapt them to physical activity by Fredriksson et al. (2018). The first level (level 1) has to do with knowing that physical activity is beneficial for health and physical inactivity is harmful to health. The second level (level 2) of knowledge involves knowing that a lack of physical activity can lead to particular diseases, such as cardiovascular disease (e.g., heart attack). The third level (level 3), knowledge of how much physical activity (frequency, duration, intensity), is needed to gain health benefits. Furthermore, the fourth level (level 4), is knowledge that involves people agreeing and understanding that their physical (in)activity poses significant risks or benefits to their health.

Reinforcement was the psychological construct that obtained the lowest levels, evidencing that eHealth does not impact this aspect the most. One possible explanation is that most of the applications have been used in free mode that does not include the features that allow sharing the results of the exercise performed. This mode, then, limits the possibility of interacting or exchanging information with other people, whether they are close (e.g., family, friends) or not (e.g., other users). Furthermore, it should be taken into account that, in times of confinement, the possibility of practicing a physical activity in groups was prohibited and limiting, even more with the possibility of having asocial reinforcement regarding the exercise practiced. A possible solution would be to incorporate this function in the free modality so that, during a period of confinement, one can interact and share the experiences of physical activity. According to recent research, more individuals were socially connected through digital technology during confinement (Ammar et al., 2020b). Taking these results into account, one could take advantage of the fact that people who share their results in eHealth exercise and physical activity tend to practice more physical activity (Consolvo et al., 2006).

As far as the Theory of Planned Behavior is concerned, attitudes, the perceived social pressures to perform the behavior (subjective norm), and control of perceived behavior determine the intention of the behavior—the proximate determinant of behavior (Ajzen and Fishbein, 1980). The results of this study showed that eHealth users perceived mainly a more important effect on their intentions and behavioral attitude toward physical activity during confinement.

The intention to engage in physical activity is one of the determinants of whether or not an individual engages in that behavior (Chatzisarantis et al., 2019). The intention is reflected in a person’s will and in the effort that such individual plans to exert to carry out the behavior. Therefore, if eHealth succeeds in increasing intentions toward physical activity, eHealth users will be more likely to engage in that behavior (Tuman and Moyer, 2019). Therefore, if someone had a clear intention to use the application to exercise during confinement, it is likely that that person would have done so.

The other psychological construct that was influenced by the use of eHealth for physical activity was attitude, which represents an individual’s positive or negative assessment of performing a behavior. This effect could be explained by behavioral beliefs, which refer to the perceived consequences of carrying out a specific action and our assessment of each of these consequences. When practicing a physical activity via eHealth, the person evaluates the consequences of each of these beliefs. Common behavioral beliefs for physical activity include believing that it improves fitness or health, improves physical appearance, is fun and enjoyable, increases social interactions, and improves psychological health (Bellows-Riecken et al., 2013). For example, people may have a negative attitude toward walking in the neighborhood during confinement but rather have a positive attitude toward physical activity in their home.

In contrast, subjective norms or the probability that individuals and relevant reference groups approve or disapprove physical activity during confinement presented the lowest level. Subjective norm reflects the perceived social pressure that individuals feel to perform or not perform a behavior. Subjective norm is believed to be a function of normative beliefs, which are determined by the perceived expectations of other significant people (e.g., family, friends, physician) or groups (e.g., classmates, teammates) and by the individual’s motivation to meet the expectations of these significant people. For example, an individual may feel that his or her friends think he or she should exercise three times a week. However, this person may not be inclined to act on these perceived beliefs. This result could be due to two conditions, confinement and spatial distancing, combined with the absence of interaction among eHealth users. These conditions may not be conducive to the development of subjective norms related to physical activity.

The third group of hypotheses considered that self-efficacy and attitudes would be positively associated with physical activity levels. This hypothesis was partially confirmed. The relationship between these psychological constructs and physical activity measured by questionnaires was mixed. When the total minutes of each intensity measured with the IPAQ (i.e., walk, moderate, vigorous) were taken into account, self-efficacy was negatively associated with walking levels, and the efficacy of automaticity positively predicted vigorous physical activity levels. This result could be explained by the type of physical activity proposed by the eHealth, fitness. The proposed exercises include abdominals, arm exercises, and no major movements such as walking or running, two of the main types of moderate physical activity (Prince et al., 2019). In contrast, when considering levels of vigorous physical activity in segments of at least 20 min over 3 days, people with high self-efficacy, high behavioral attitude, and high intention advocacies outnumbered those with ambivalence and low advocacy in reaching those levels. These results demonstrate that physical activity levels measured by IPAQ should not only consider the total minutes of each type of physical activity but also how these minutes accumulate (Di et al., 2017).

The fourth hypothesis, a positive relationship between automaticity of habits toward eHealth and the levels of physical activity, was confirmed. Several authors have proposed that the cognitive processes that guide healthy behaviors such as physical activity should include not only intentional processes but also automatic processes such as habits (Evans and Frankish, 2009; Gardner et al., 2011; Marteau et al., 2012). However, to our knowledge, these elements had not been studied in eHealth for exercise and physical activity. Therefore, this study would be the first to address this issue. Within the theoretical framework of habits, frequency (Verplanken, 2006), context stability (Wood and Neal, 2016), and automaticity (Gardner, 2012) have been described as the three pillars of habits (Orbell and Verplanken, 2015). In this research, the frequency and automaticity of eHealth were shown to be positively related to its adoption and levels of physical activity.

Similarly, confinement “forced” the stability of exercise and physical activity practice contexts. At the same time, at the beginning of this period, confinement may have been experienced as a moment of disruption of daily life (Wood et al., 2005). Nevertheless, as the days went by 55 in France, it became a habitual context.

In this way, the incorporation of the use of eHealth for exercise and physical activity acquired automatic properties that facilitated the habit development of these technologies (Larose, 2015) and the practice of physical activity. These elements combined together are highly conducive to habit formation (Lally et al., 2010). Thus, these results demonstrated that higher levels of habit automaticity of using eHealth for physical activity mean higher levels of physical activity (Gardner et al., 2011). Our results also showed, as pointed out by Bargh (1994), that the unidimensional definition of automaticity is no longer tenable. Using eHealth for exercise and physical activity requires the intention to use it, but it also could develop automatic properties (at least for the regular eHealth user). In this study, the property of the automaticity of eHealth use for exercise and physical activity that had the most significant influence on behaviors was efficiency. Once eHealth for exercise and physical activity use has started, eHealth users can present a lack of need for attentional resources (Bargh, 1994), making the eHealth utilization very efficient. This result translated into more minutes of physical activity (Boiché et al., 2016), confirming the importance of considering automatic properties of habits in eHealth for exercise and physical activity behaviors.

### Limitations

Although this study is a first insight into the effects of eHealth for exercise and physical activity on the motivations, psychological mechanisms, and behavior in this COVID-19 pandemic, this study is not exempt from some limits that should be taken into account when interpreting our results. The use of the Internet to distribute and complete questionnaires could represent a bias in the sample. In fact, the demographics of the respondents to our online survey may differ from that of the sample population as a whole. For example, Sue and Ritter (2012) note that an online population may contain a higher proportion of individuals of higher socioeconomic status than the total population and does not reflect the population as a whole. Another aspect that should be considered is the absence of analysis of the quality of eHealth. For example, videos or applications may have had different qualities in terms of their content, which could translate into factors that contribute to or undermine motivations. Despite widespread Internet access, the use of physical activity eHealth is still limited to one age group, so Internet access alone is not enough, and how and where information about eHealth is presented, organized, and disseminated are equally important. Currently, limited information exists about how to operationalize content and strategies best to maximize eHealth use among underserved populations (e.g., over 50 years old or less qualified population). The measurement of physical activity behavior has been via questionnaires that may present cognitive biases due to their subjectivity (Silfee et al., 2018). However, the context of the COVID-19 pandemic has not made possible the measurement of behaviors with objective methods (e.g., accelerometers).

## Conclusion

In conclusion, the use of a physical activity eHealth modified the practice of physical activities of its users during confinement. Indeed eHealth users affected mainly the levels of vigorous physical activity. This change in behavior could also be due to the positive impact that eHealth would have on self-efficacy and attitudes toward physical activity. Particular attention should be paid to the automaticity of eHealth use habits for physical activity, as people who had high levels of these properties practiced more physical activity. The eHealth for physical activity and exercise should incorporate information to guide people on recommended levels, frequency, and intensity of physical activity for health to go beyond the simple notion of knowing that physical activity is “good” for health. Similarly, in the context of confinement, eHealth should enable a more significant interaction between users, and the effects of eHealth could be explored to reduce the time people spend sitting down. Our results appear to be encouraging regarding the use of eHealth for exercise and physical activity. However, its effectiveness remains to be demonstrated. Besides, our study confirms that eHealth for exercise and physical activity incorporates only a few behavioral change techniques. The developers of these technologies should consider including more behavior change techniques to more accurately increase physical activity levels.

## Data Availability Statement

The raw data supporting the conclusions of this article will be made available by the authors, without undue reservation.

## Ethics Statement

The studies involving human participants were reviewed and approved by Commission Nationale de l’Informatique et des Libertés. French methodological reference MR-001. This statement was in accordance with the Declaration of Helsinki and the participants were requested to be honest and as accurate as possible in their responses. The patients/participants provided their written informed consent to participate in this study.

## Author Contributions

GM contributed to research design, data collection, management and coordination responsibility for the research activity planning and execution, and manuscript writing. MB contributed to conceptualization, formulation and evolution of overarching research goals and aims, and manuscript writing. FB and EG contributed to critical review, commentary, and revision. All authors contributed to the article and approved the submitted version.

## Conflict of Interest

The authors declare that the research was conducted in the absence of any commercial or financial relationships that could be construed as a potential conflict of interest.
